# A case of gestational gigantomastia in a 37-years-old woman associated with elevated ANA: a casual linkage?

**DOI:** 10.11604/pamj.2017.27.167.11281

**Published:** 2017-07-04

**Authors:** Nicola Zingaretti, Fabrizio De Biasio, Michele Riccio, Nastassia Nardini, Laura Mariuzzi, Pier Camillo Parodi

**Affiliations:** 1 Department of Plastic and Reconstructive Surgery, Breast Unit, University of Udine, Italy; 2 Department of Reconstructive Plastic Surgery-Hand Surgery, Breast Unit, AOU “Ospedali Riuniti”, Ancona, Italy; 3 Section of Surgical Pathology, Department of Medical and Biological Sciences, University Hospital of Udine, Italy

**Keywords:** Gigantomastia, pregnancy, reduction mammoplasty, breast hypertrophy, undifferentiated connective tissue disease

## Abstract

Hypertrophy of the breast (macromastia and gigantomastia) is a rare medical condition of the breast connective tissues. The etiology of this condition is still not clear; rarely, gigantomastia has been reported to develop in the setting of an autoimmune illness. We reported a case of a 37-years-old woman with undifferentiated connective tissue disease of 2-years duration presented with enlargement of breasts. The breast enlargment started at 5 months of gestation. She successfully underwent reduction mammoplasty with free nipple graft. In the succeeding months the level of antinuclear ANA remained stable. It is uncertain whether a positive antinuclear antibodies in gigantomastia is a casuative agent or an effect.

## Introduction

The increase of breast size in puberty and pregnancy is a physiological event. Breast hypertrophy is a benign progressive enlargement, which can occur in both breasts or only in one breast. Gigantomastia is a rare breast condition characterised by rapid, diffuse and excessive breast hypertrophy (> 1,5 kg). The extremely rapid growth of the breasts can result in intense heat; the enlargement can cause muscular discomfort and over-stretching of the skin envelope, which can lead in some cases to ulceration. The swelling can suppress the milk supply, pinching off the milk ducts, and leading to mastitis. It may be associated with puberty, pregnancy, induced by drugs, in a context of immune-mediated diseases or without an associated cause (idiopathic).

## Patient and observation

**Figure 1:**
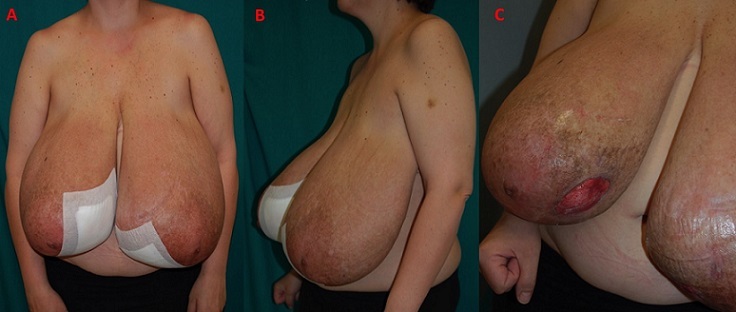
Frontal view (A) and side views (B,C) in a 32-week pregant patient with gigantomastia. The patient presents with peau d’orange texturing of breasts, ulcerations and dilated veins on the upper thorax

A 37-years-old woman presented at29 weeks gestation with marked bilateral breast swelling;this enlargement started at 23 weeks’ gestation and continued to increase rapidly as the pregnancy progressed, complicated by infection skin atrophy, marked venous engorgement, ulcerations, necrosis and subsequent hemorrhage. She reported that this was the first time she had experienced excessive breast enlargement; this had not occurred in her previous pregnancy (4 years ago). During her hospitalization, breasts continued to increase in size (bra size passed from fifth to tenth measure),with skin atrophy, necrosis and multiple ulcers measuring 2-5 cm in diameter (Figure 1). The patient was complaining on mastalgia, severe back pain, breathing difficulties, moving difficulties and pain near ulcerations.

Past surgical history included only a cesarean and appendicectomy (when she was 14 yo). She had no familial history of breast disease. Her medical history is notable for mild Raynaud´s phenomenon and recurrent arthralgias (most commonly of the hands and feet), but she had never been treated for arthralgias. She had been diagnosed withundifferentiated connective tissue disease three years before. Laboratory assays detected the presence of antinuclear ANA (1:2560, homogenous and nucleolar) and elevated anti-dsDNA. Tests for antibodies to anti-ENA and for antiphospholipid (lupus anticoagulant and anticardiolipin), anticentromere, anti-Scl-70, and antiplatelet antibodies were negative, as was a Coombs test.

Complete blood count, erythrocyte sedimentation rate, C-reactive protein, transaminase (GPT and GOT), γ-GT, thyroid stimulating hormone, follicle-stimulating hormone, luteinizing hormone, estradiol, and prolactin were all within normal limits. An echocardiogram, chest X-ray, and abdominal scan showed no abnormalities. Nailfold digital capillaroscopy revealed minor capillary changes not specific for scleroderma nor capillaroscopic alterations suggestive of systemic sclerosis. Ultrasound demonstrated diffused breast edema involving the cutaneous tissue, signs of inflammation, lymphatic dilatation, parenchymal and skin edema and couldn’t rule out underlying malignancy. She then underwent bilateral incisional breast biopsies to exclude carcinoma. The biopsies showed no histological evidence of malignancy.

Her condition significantly improved, but she noted enlargement of breasts (at thight level); the nipple tip- lower clavicle distancewas measured at 46,5 cm on the right and 48 cm on the left;nipple tip– inframammary fold distance (along the mammary meridian) was 22,5 cm on the right and 24 cm on the left (measured with an ordinary ruler or caliper tool). The patient became an antibiotics therapy, dialy dressing, haematinics and nanalgesics. At the beginning of the 38 weeks of gestation, the gynecologists made an indication for a delivery with caesarean section. Furthermore, on request of the patient, sterilization was made. She was evaluated by plastic surgery at 3 weeks and 6 weeks postpartum for a bilateral breast reduction.

**Figure 2:**
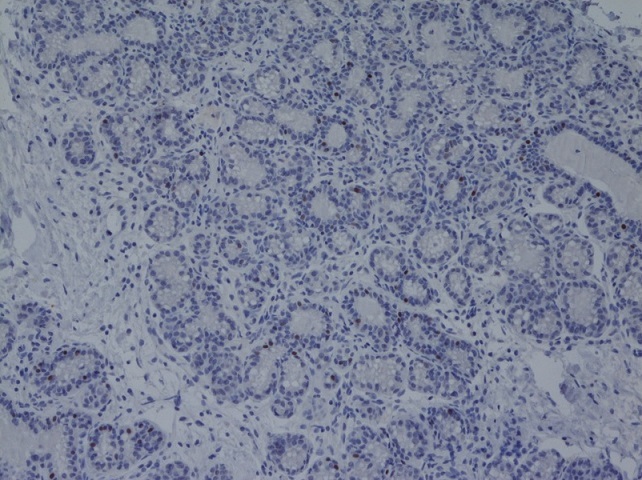
Biopsy of mammary gland with edema, abundant proliferation of stromal loose connective tissue, myxoid degeneration of the stroma, lobular ductal hyperplasia and cystic dilation of breast ducts.

**Figure 3:**
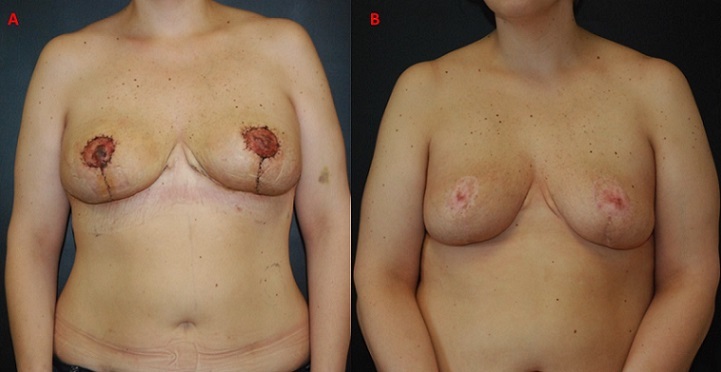
Postreduction mammoplasty with free nipple graft 2 months after surgery (A) and 12 months after surgery (B)

Six weeks later, bilateral reduction mammoplasty (3,23 kg and 4,20 kg of tissue were removed from the right and left breasts, respectively) with free nipple areola graft technique was performed. The biopsies showed stromal fibrosis, edema, myxoid degeneration of the stroma, lobular ductal hyperplasia and cystic dilation of breast ducts. There was no chronic inflammatory cellular infiltrate and histologic evidence of malignancy (Figure 2). Estrogen and progesterone receptors were negative. These analyses were performed twice in order to confirm the initials results. The patient was followed for 12 months with no sign of recurrence or breast carcinoma found on physical examination and sonography (Figure 3). Written informed consent was obtained from patient.

## Discussion

The pathophysiology of gigantomastia is still unknow; although no precise mechanism is known for this rare clinical entity, theories such as end-organ hyper-sensitivity, autoimmune stimulation antibodies, high IGF-1 and hyperprolactinemia have been proposed. Several cases of gigantomastia have been described in association with autoimmune disorders, such as systemic lupus erythematosus, myasthenia gravis, Grave’s disease, chronic arthitis, psoriasis and Hashimoto’s thyroiditis [1–4].

Undifferentiated connective tissue disease (UCTD) is a systemic autoimmune disease that is characterized by the presence of signs and symptoms that are suggestive of a connective tissue disease but does not fulfill the criteria for any defined connective tissue disease. This disorder is recognized as a distinct entity in the classification of connective tissue diseases and is characterized by a mild clinical profile and a simplified autoantibody profile and could be considered as a specific clinical entity and not early or incomplete phase of defined Connective Tissue Disease (such as systemic lupus erythematosus, rheumatoid arthritis and antiphospholipid syndrome).

The most common clinical manifestations are non-erosive arthritis, Raynaud’s phenomenon without swollen hands, sclerodactily or nailfold capillaroscopic abnormalities suggestive of sistemic sclerosis, sicca symptoms and leucopenia. The sierological profile is positive for antinuclear antibodies (ANA), generally with a single autoantibody specifity (anti-Ro/SSA or anti-RNP) and only rarely disease-specific autoantibodies (usually anti-dsDNA antibodies). The analysis of the autoantibody specificities during the follow-up showed that UCTD patients have a stable profile and do not develop new specificities [5].

Regarding the effect of pregnancy on the course of UCTD, the data of Spinillo et al suggest that 25% of patients experience a significant rheumatic disease flare during pregnancy; pregnant patients with newly diagnosed UCTD are at increased risk of impared intrauterine growth, prematurity and small for gestional age infants [6]. Castellino et al. reported pregnancy in patients with UTCD appear to be a risk factor for flare or evolution into definite CTD [7]. Our patient had been diagnosed with UCTD three years before, which resulted in stable disease (same symptoms) for three years, and it did not change during pregnancy.Furthermore, the antibody level remained stable over time. Vinicki et al. proposed that pregnancy could be a trigger forrheumatic disease emergence, suggesting that gigantomastia in pregnancy might be the result of an underlying autoimmune disorder [3]. Antinuclear antibodies (ANAs) are a diverse group of autoantibodies that target macromolecular components of the cell nucleus. These antibodies occur commonly in the sera of patients with autoimmune and rheumatic disease and bind proteins, nucleic acids and complexes of proteins and nucleic acids.

Certain drugs, infectious agent, and other environmental factors such as sunlight or silica dust may induce autoantibodies including ANA with or without development of autoimmune disease manifestations. Despite these antibodies occur in the circulation of all human beings, but the employed test is only considered “positive” if they occur at titres elevated significantly above the normal serum level. Elevated levels of ANA are found in all systemic rheumatic disease, with sometimes high, sometimes rather loose associations between a particular ANA specificity and a particular rheumatic disease. It is uncertain wheter a positive ANA in gigantomastia is a casuative agent or an effect of the gigantomastia. The formation of ANA is suggested to play some roles in physiological immune homeostasis [8].

Our patient showed a homogeneous pattern of ANA; this type of pattern of ANA was previously observed in 70% of patients with morphea. A homogeneous pattern might be associated with antibodies to native DNA or antibodies to histone and increased manifestation of autoimmunity [9]. Despite the clinical association of ANAs, the relationship of these antibodies to specific disease manifestations is often unknown because the target antigens are intracellular molecules that are highly conserved and ubiquitously expressed.

The persistence of this type of autoreactivity in the human population suggests that antinuclear antibodies may be an important component of the normal immune response. Immunological changes are known to occur within the immune system during pregnancy; ANA (mild titer) in normal pregnancy has been variously reported to range from 1-53% [7]. As suggested by Gronkowski et al about the correlation between ANA and infertility (“Antinuclear antibodies cause an inflammation in the uterus that does not allow it to be a suitable host for implantation of the embryo”) [10], the mechanism by which ANAs cause gigantomastia based on a hypothesis antinuclear antibodies cause an inflammation in the breast that lead to anabnormal proliferation of glandular tissue.

It is possible that gigantomastia is a manifestation of a UCTD flare during pregnancy? It is a casual linkage? It is possible that antinuclear antibodies cause an abnormal proliferation of breast? What´s the mechanism? We don’t think it’s a coincidence that this patient showed the extremely rapid growth of the breasts after a diagnosis of UCTD, because she didn’t present this type of complication in her first pregnancy. About surgical management, reduction mammaplasty asorgan-conserving therapy is a method of choice in a situation when no next pregnancy is expected or when the operation is performed in line with the delivery, so there is no opportunity for recidivating.

## Conclusion

This report describes the first known case of gestional gigantomastia associated with undifferentiated connective tissue disease; if there is a role of ANA in the genesis of this disease it is uncertain wheter is a casuative agent or an effect of the gigantomastia.

## Competing interests

The authors declare no competing interests.
